# Supplementary material for: Postprandial Apolipoprotein B48 is Associated with Subclinical Atherosclerosis in Patients with Rheumatoid Arthritis
Source: J Clin Med. 2020 Aug 2;9(8):2483. doi: 10.3390/jcm9082483 (PMC7465472; doi:10.3390/jcm9082483)
Supplement: Supplementary file 1 [file jcm-09-02483-s001.pdf]

Supplementary material

Postprandial Apolipoprotein B48 is Associated with Subclinical Atherosclerosis in Patients with Rheumatoid Arthritis

Natalia Mena-Vázquez [1,2], Marta Rojas-Gimenez [3,*], Francisco Gabriel Jimenez Nuñez [1,2], Sara Manrique-Arija [1,2], José Rioja [1,4], Patricia Ruiz-Limón [1,5], Inmaculada Ureña [1,2], Manuel Castro-Cabezas [6], Pedro Valdivielso [1,4,7,†] and Antonio Fernández-Nebro[1,2,4,†]

1. Instituto de Investigación Biomédica de Málaga (IBIMA), 29010, Málaga, Spain; nataliamenavazquez@gmail.com (N.M.-V.); sarama_82@hotmail.com (S.M.-A.); jose.rioja@uma.es (J.R.); patrilimon@hotmail.com (P.R.-L.); inuregar@gmail.com (I.U.); valdivielso@uma.es (P.V.); afnebro@gmail.com (A.F.-N.)
2. UGC de Reumatología, Hospital Regional Universitario de Málaga, 29009, Málaga, Spain
3. UGC de Reumatología, Instituto Maimónides de Investigación Biomédica de Córdoba (IMIBIC). Hospital Universitario Reina Sofia, Córdoba, Spain; cortesfra@gmail.com (F.G.J.N.)
4. Departamento de Medicina y Dermatología, Universidad de Málaga, Málaga, Spain.
5. Unidad de Gestión Clínica de Endocrinología y Nutrición, Hospital Clínico Virgen de la Victoria, 29010, Málaga, Spain
6. Department of Internal Medicine, Franciscus Gasthuis & Vlietland, Rotterdam, The Netherlands; m.castrocabezas@franciscus.nl
7. UGC de Medicina Interna, Hospital Universitario Virgen de la Victoria, Universidad de Málaga, Málaga, Spain.

* Correspondence: rojasgimenezm@gmail.com; Tel.: +xx-xxxx-xxx-xxxx

Table S1. Lipid profile and carotid ultrasound in control group according to cIMT.

Variable	Control group with IMT >p90 *n* = 9		Control group with IMT ≤p90 *n* = 31		Control group with IMT >p90 vs with IMT ≤p90 *p*-value	
	**Fasting**	**Postprandial**	**Fasting**	**Postprandial**	**Fasting**	**Postprandial**
**Fasting lipid profile**						
Total cholesterol (mg/dl), median (IQR)	190.0 (171.1–226.5)	189.0 (166.5–220.5)	202.0 (176.0–236.0)	201.5 (167.7–227.0)	0.656	0.678
LDL cholesterol (mg/dl), median (IQR)	110.0 (83.5–148.0)	108.0 (75.5–131.5)	120.0 (96.0–142.0)	108.5 (83.0–128.0)	0.667	0.638
HDL cholesterol (mg/dl), median (IQR)	56.0 (45.5–74.0)	53.0 (46.5–68.5)	60.0 (48.0–71.0)	58.0 (45.0–66.0)	0.762	0.830
Triglycerides (mg/dl), median (IQR)	72.0 (54.5–140.5)	116.5 (67.7–206.7)	86.0 (60.0–128.0)	136.0 (97.2–170.0)	0.545	0.473
Chylomicrons (triglycerides), median (IQR)	15.2 (5.3–42.9)	51.2 (15.3–78.2)	17.0 (7.5–45.3)	54.3 (32.4–80.7)	0.831	0.473

Chylomicrons (cholesterol), median (IQR)	5.5 (2.6–22.4)	29.1 (5.5–36.8)	11.0 (7.5–45.3)	14.1 (8.7–32.9)	0.509	0.497
VLDL (triglycerides), median (IQR)	13.6 (8.5–43.7)	24.2 (11.2–57.1)	20.7 (9.8–31.1)	25.0 (17.9–38.1)	0.935	0.911
VLDL (cholesterol), median (IQR)	5.9 (1.6–14.1)	7.1 (4.0–15.6)	5.7 (2.8–8.3)	7.4 (3.9–10.6)	0.730	0.543
ApoB48, median (IQR)	5.2 (4.6–8.0)	14.4 (9.4–15.6)	8.3 (6.4–11.8)	13.9 (11.7–17.2)	0.116	0.274
ApoB total, median (IQR)	101.9 (80.0–108.0)	94.1 (74.0–105.1)	96.8 (82.4–111.2	92.9 (76.7–104.0)	0.975	0.846
**Increased postprandial blood lipids**						
Triglycerides (mg/dl), median (IQR)		42.7 (16.1–128.8)		48.4 (32.2–75.8)		0.709
Chylomicrons (triglycerides), median (IQR)		29.3 (11.8–135.3)		33.5 (13.1–64.0)		0.289
VLDL (triglycerides), median (IQR)		9.8 (2.2–22.4)		8.4 (2.0–17.7)		0.975
ApoB48, median (IQR)		6.7 (3.3–9.9)		6.9 (2.2–9.8)		0.920

* $p < 0.005$ fasting vs postprandial value. Abbreviations: cIMT, carotid intima media thickness; LDL, low-density lipoprotein; HDL, high-density lipoprotein; TG, triglycerides; VLDL, very-low-density lipoprotein.

Table S2. Lipid profile and carotid ultrasound in RA patients according to cIMT excluded the men.

**Variable**	**RA with IMT >p90 n=6**		**RA with IMT ≤p90 *n* = 29**		**RA with IMT >p90 vs RA with IMT ≤p90 *p***	
	**Fasting**	**Postprandial**	**Fasting**	**Postprandial**	**Fasting**	**Postprandial**
**Fasting lipid profile**						
Total cholesterol (mg/dl), median (IQR)	234.5 (212.7–255.7)	210.0 (195.2–252.5)	212.0 (185.5–227.5)	200.0 (177.0–228.0)	0.176	0.235
LDL cholesterol (mg/dl), median (IQR)	139.0 (133.0–165.5)	116.0 (105.7–145.0)	122.0 (103.5–140.5)	110.0 (94.0–129.5)	0.164	0.312
HDL cholesterol (mg/dl), median (IQR)	66.5 (60.2–78.2)	62.5 (55.7–72.2)	66.0 (53.5–80.5)	62.0 (52.5–77.0)	0.749	0.783
Triglycerides (mg/dl), median (IQR)	112.0 (83.5–165.7)	195.0 (125.2–326.5)	74.0 (63.0–105.0)	111.0 (83.0–163.5)	0.021	0.042
Chylomicrons (triglycerides), median (IQR)	52.7 (16.6–64.9) *	77.7 (22.9–195.4) *	13.8 (8.7–20.9)	34.2 (21.7–48.9)	0.011	0.052

Chylomicrons (cholesterol), median (IQR)	13.4 (8.0–23.3)	15.5 (5.7–33.2)	8.5 (6.0–10.7)	14.3 (7.2–23.2)	0.093	0.782
VLDL (triglycerides), median (IQR)	27.2 (21.9–42.4)	37.3 (17.3–60.0)	13.8 (9.7–23.1)	20.7 (12.9–38.7)	0.011	0.098
VLDL (cholesterol), median (IQR)	5.9 (4.9–10.7)	8.8 (3.4–12.7)	2.7 (1.9–5.4)	3.9 (2.6–8.9)	0.014	0.048
ApoB48, median (IQR)	7.5 (5.5–12.1) *	23.7 (10.0–31.1) *	7.0 (6.1–10.2)	13.4 (10.4–18.4)	0.685	0.046
ApoB total, median (IQR)	104.0 (98.9–120.8)	106.3 (89.8–114.0)	94.5 (79.2–105.5)	91.9 (75.3–103.0)	0.093	0.062
**Increased postprandial blood lipids**						
Triglycerides (mg/dl), median (IQR)		63.7 (16.2–140.0)		35.7 (17.1–61.6)		0.279
Chylomicrons (triglycerides), median (IQR)		46.4 (11.0–147.0)		20.0 (10.2–34.7)		0.042
VLDL (triglycerides), median (IQR)		11.8 (6.4–22.0)		7.0 (3.0–16.3)		0.454
ApoB48, median (IQR)		13.0 (6.2–16.4)		6.7 (3.4–8.4)		0.032

* $p < 0.005$ fasting vs postprandial value. Abbreviations: cIMT, carotid intima media thickness; LDL, low-density lipoprotein; HDL, high-density lipoprotein; TG, triglycerides; VLDL, very-low-density lipoprotein.
